# UNDERSTANDING VOTING TRENDS AMONG AGING AMERICANS

**DOI:** 10.1093/geroni/igae098.0307

**Published:** 2024-12-31

**Authors:** Goldie Byrd, Capri Foy, Allison Caban-Hol, TanYa Gwathmey, Patricia Smyre, Kelvin Williams

**Affiliations:** Wake Forest University School of Medicine, Winston-Salem, North Carolina, United States; Wake Forest University School of Medicine, Winston-Salem, North Carolina, United States; Wake Forest University School of Medicine, Winston-Salem, North Carolina, United States; Wake Forest University School of Medicine, Winston-Salem, North Carolina, United States; Wake Forest University School of Medicine, Winston-Salem, North Carolina, United States; Wake Forest University School of Medicine, Winston-Salem, North Carolina, United States

## Abstract

The right to vote, also known as “the franchise”, is a fundamental right of United States (US) citizens, and is essential to preserve the democratic republic of the US. However, voting often requires significant planning, time and effort, and there are considerable and persistent challenges and threats to voting for several demographic groups in the US, including older adults. In this analysis, we will examine voting trends in this population in four recent general Presidential Elections (2008, 2012, 2016, and 2020) using data from the Current Population Survey (CPS). Specifically, we will examine self-reported voting percentages among noninstitutionalized older US adults (ages 60 years or older) who report having disabilities compared to older adults without disabilities, after adjustment for demographic variables. In addition, we will compare engagement in early voting, as well as voting in person or via mail, among older adults with and without disabilities. Lastly, we will examine whether these results are moderated by race, gender or other demographic factors (e.g. education). Results will be discussed in the context of the extant literature, as well as the history of legislative facilitators and barriers to voting for American adults with disabilities. Finally, we discuss voting trends among adults with disabilities for the 2024 Presidential Election, and we provide suggestions for future education and research and additional facilitators to enhance voting rates for older Americans with or without disabilities.

